# Supplementary material for: Prodynorphin and Proenkephalin in Cerebrospinal Fluid of Sporadic Creutzfeldt–Jakob Disease
Source: Int J Mol Sci. 2022 Feb 12;23(4):2051. doi: 10.3390/ijms23042051 (PMC8877714; doi:10.3390/ijms23042051)

SUPPLEMENTARY MATERIALS

Supplementary results

In the whole cohort age did not influence levels of PDYN peptide [SVG...LAR] (r=0.071, p=0.509), PDYN peptide [FLP...STR] (r=0.178, p=0.097), PENK peptide [DAE...LLK] (r=-0.160, p=0.198), PENK peptide [FAE...YSK] (r=0.063, p=0.560). Similarly, there was no association between sex and levels of PDYN peptide [SVG...LAR] (p=0.434), PDYN peptide [FLP...STR] (p=0.476), PENK peptide [DAE...LLK] (p=0.444), PENK peptide [FAE...YSK] (p=0.449).

PENK peptide [DAE...LLK] was significantly lower in CJD than controls even after age-adjustment (β=-0.374, p=0.002), while the other peptides did not differ between CJD and controls. Mean PENK but not mean PDYN was significantly lower in CJD compared to controls (p=0.001).

After age-adjustment, the MV2K group showed significantly lower PDYN [SVG...LAR] levels compared to the VV2 (β=-0.304, p=0.044) group and significantly lower PDYN [FLP...STR] levels than the VV2 (β=-0.291, p=0.042) and MM(V)1 (β=-0.251, p=0.048) groups. PDYN peptides were significantly lower in MV2K patients than in controls ([SVG...LAR]: β=-0.362, p=0.023, [FLP...STR]: β=-0.242, p=0.049). Mean PDYN was lower in MV2K compared to VV2 (p=0.025) and MM(V)1 (p=0.039) and controls (p=0.027). PENK [DAE...LLK] and [FAE...YSK] peptide levels did not differ among the most prevalent CJD subtypes. However, the PENK [DAE...LLK] peptide was significantly decreased in each CJD subtype compared to controls after age-adjustment (MM(V)1: β=-0.518, p=0.001, VV2: β=-0.382, p=0.026, MV2K: β=-0.308, p=0.041).

Table S1. Diagnostic accuracy (AUC values) of CSF PDYN and PENK peptides in the distinction between sCJD subtypes and controls.

AUC	PDYN [SVG…LAR] L/H ratio	PDYN [FLP…STR] L/H ratio	PENK [DAE…LLK] L/H ratio	PENK [FAE…YSK L/H ratio
**sCJD all vs. controls**	0.605±0.068	0.479±0.067	0.768±0.061	0.516±0.073
**sCJD MM(V)1 vs. controls**	0.622±0.098	0.583±0.100	0.844±0.077	0.575±0.102
**sCJD VV2 vs. controls**	0.515±0.097	0.426±0.096	0.741±0.089	0.482±0.103
**sCJD MV2K vs. controls**	0.704±0.088	0.619±0.095	0.711±0.100	0.469±0.106
**sCJD MM(V)1 vs. VV2**	0.621±0.109	0.638±0.103	0.563±0.109	0.558±0.108
**sCJD MM(V)1 vs. MV2K**	0.602±0.107	0.538±0.110	0.400±0.109	0.362±0.104
**sCJD VV2 vs. MV2K**	0.704±0.088	0.619±0.098	0.711±0.100	0.469±0.106

Table S2. Median levels and interquartile ranges of CSF biomarkers in sCJD patients.

Diagnosis	sCJD
**N**	63
**t-tau (pg/ml)**	4501 (2270-10400)
**NfL (pg/ml)**	8582 (4943-12225)
**14-3-3 (AU/ml)**	77450 (29200-156350)
**YKL-40 (pg/ml)**	368 (274-487)

Table S3.

Diagnosis	MM(V)1	VV2	MV2K
**N**	28	20	15
**Age at LP (years ±SD)**	70.39 ±9.57	71.70 ± 7.90	67.93 ± 6.05
**Female (%)**	53.6	35.0	66.7
**Time from symptom onset to LP (months ±SD)**	1.95 ± 1.25	3.28 ± 1.13	6.19 ± 3.80
**Survival (months ±SD)**	3.25 ± 1.47	5.57 ± 2.08	17.46 ± 14.86
**Presenting symptom(s)* (%):**			
**Cognitive**	16 (57.1)	5 (25.0)	9 (60.0)
**Ataxia**	7 (25.0)	18 (90.0)	6 (40.0)
**Oculomotor**	0 (0)	3 (15.0)	1 (6.7)
**Psychiatric**	3 (10.7)	1 (5.0)	1 (6.7)
**Extrapyramidal**	2 (7.1)	0 (0)	1 (6.7)
**Pyramidal**	2 (7.1)	0 (0)	0 (0)
**CSF 14-3-3 protein (positive/tested, %)**	26/28 (92.8)	20/20 (100)	5/15 (33.3)
**EEG PSWCs (positive/tested, %)**	17/25 (68.0)	3/17 (17.6)	0/12 (0)
**Typical MRI (positive/tested, %)**	17/21 (80.9)	12/14 (85.7)	8/9 (88.9)
**CSF prion RT-QuIC (positive/tested, %)**	27/28 (96.4)	20/20 (100)	14/15 (93.3)

*Symptoms occurred in less than 2 patients in the overall sCJD cohort were not included.

Figure S1. Correlations between CSF PDYN and PENK peptides and CSF biomarkers of neurodegeneration. Significant spearman rank correlations between CSF PDYN and PENK peptides and CSF t-tau, 14-3-3 protein and NfL are shown in panels A-E.

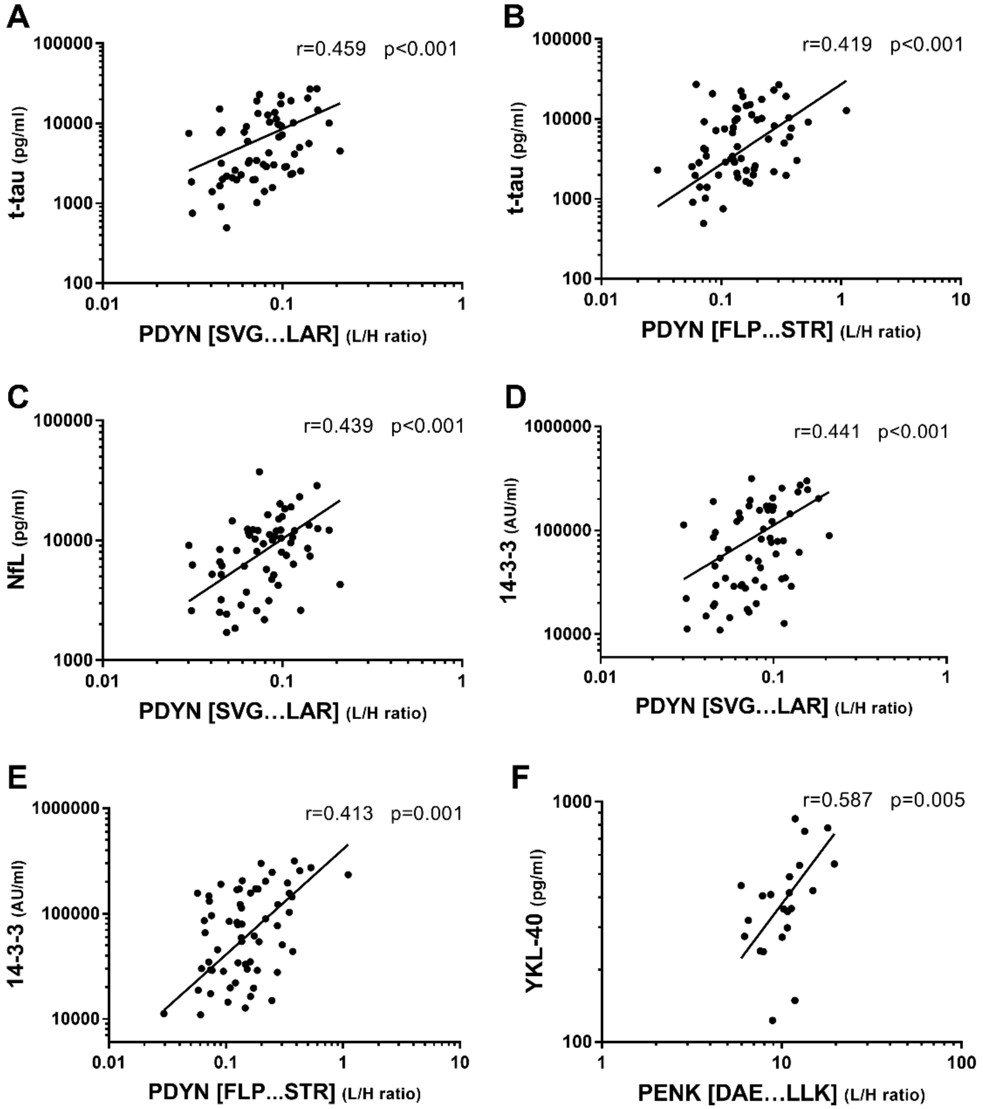

Supplementary Methods

200 microliters of CSF sample were mixed with 12 µL internal standard solution containing heavy labelled peptides and 20 µL 1M triethylammonium bicarbonate. Reduction and alkylation were conducted with 20 µL 1M tris(2-carboxyethyl) phosphine and 2 µL 200 mM chloroacetamide for 10 min at 95°C and 400 rpm. Samples were digested with 10 µL trypsin/Lys-C solution (0.1 µg/µL) for 18 hours at 37°C and 400 rpm. The reaction was stopped by adding trifluoroacetic acid (TFA) to a final concentration of 1%. Samples were fractionated using in-house prepared strong cation exchange STAGE tips. Peptides were eluted with an increasing concentration of ammonium acetate as follows: 125mM ammonium acetate/ 20% acetonitrile/ 0.5% formic acid (fraction 1), 160mM ammonium acetate/ 20% acetonitrile/ 0.5% formic acid (fraction 2), 225mM ammonium acetate/ 20% acetonitrile/ 0.5% formic acid (fraction 3), 300mM ammonium acetate/ 20% acetonitrile/ 0.5% formic acid (fraction 4), 450 mM ammonium acetate/ 20% acetonitrile/ 0.5% formic acid (fraction 5) and 5% ammonium hydroxide/ 80% acetonitrile (fraction 6). Eluates of fractions one, two and five were dried by vacuum centrifugation and dissolved in 27.5 µL 0.5% TFA/6% ACN for MS analysis. Further MRM methods are provided in the Table S1.

Table S4: MRM methods

Precursor mass	Product ion mass	RT (min)	Peptide	DP	EP	CE	CXP	ST1
**Fraction 1**								
861.39	960.54	7.6	sp|P01210|PENK_HUMAN.DAEEDDSLANSSDLLK.+2y9.light	90	11	42.9	49	-22
816.39	776.41	7.6	sp|P01210|PENK_HUMAN.DAEEDDSLANSSDLLK.+2y7.light	90	11	42.9	41	-22
816.39	316.11	7.6	sp|P01210|PENK_HUMAN.DAEEDDSLANSSDLLK.+2b3.light	90	11	42.9	20	-22
865.40	968.55	7.6	sp|P01210|PENK_HUMAN.DAEEDDSLANSSDLLK.+2y9.heavy	90	11	42.9	49	-22
865.40	784.43	7.6	sp|P01210|PENK_HUMAN.DAEEDDSLANSSDLLK.+2y7.heavy	90	11	42.9	41	-22
865.40	316.11	7.6	sp|P01210|PENK_HUMAN.DAEEDDSLANSSDLLK.+2b3.heavy	90	11	42.9	20	-22
879.89	1227.50	6.1	sp|P01210|PENK_HUMAN.FAEALPSDEEGESYSK.+2y11.light	90	11	43.7	55	-22
879.89	348.16	6.1	sp|P01210|PENK_HUMAN.FAEALPSDEEGESYSK.+2b3.light	90	11	43.7	21	-22
879.89	419.19	6.1	sp|P01210|PENK_HUMAN.FAEALPSDEEGESYSK.+2b4.light	90	11	43.7	24	-22
883.90	1235.52	6.1	sp|P01210|PENK_HUMAN.FAEALPSDEEGESYSK.+2y11.heavy	90	11	43.7	55	-22
883.90	348.16	6.1	sp|P01210|PENK_HUMAN.FAEALPSDEEGESYSK.+2b3.heavy	90	11	43.7	21	-22
883.90	419.19	6.1	sp|P01210|PENK_HUMAN.FAEALPSDEEGESYSK.+2b4.heavy	90	11	43.7	24	-22
**Fraction 2**								
**Precursor mass**	**Product ion mass**	**RT (min)**	**Peptide**	**DP**	**EP**	**CE**	**CXP**	**ST1**
618.81	1050.51	5.5	sp|P01213_1|PDYN_HUMAN.SVGEGPYSELAK.+2y10.light	90	10	32.2	53	-22
618.81	864.45	5.5	sp|P01213_1|PDYN_HUMAN.SVGEGPYSELAK.+2y8.light	90	10	32.2	44	-22
618.81	807.42	5.5	sp|P01213_1|PDYN_HUMAN.SVGEGPYSELAK.+2y7.light	90	10	32.2	42	-22
618.81	432.73	5.5	sp|P01213_1|PDYN_HUMAN.SVGEGPYSELAK.+2y8+2.light	90	10	32.2	25	-22
622.82	1058.52	5.5	sp|P01213_1|PDYN_HUMAN.SVGEGPYSELAK.+2y10.heavy	90	10	32.2	53	-22
622.82	872.46	5.5	sp|P01213_1|PDYN_HUMAN.SVGEGPYSELAK.+2y8.heavy	90	10	32.2	45	-22
622.82	815.44	5.5	sp|P01213_1|PDYN_HUMAN.SVGEGPYSELAK.+2y7.heavy	90	10	32.2	42	-22
622.82	436.73	5.5	sp|P01213_1|PDYN_HUMAN.SVGEGPYSELAK.+2y8+2.heavy	90	10	32.2	25	-22

Fraction 5								
Precursor mass	Product ion mass	RT (min)	Peptide	DP	EP	CE	CXP	ST1
446.76	745.45	6.2	sp|P01213_1|PDYN_HUMAN.FLPSISTK.+2y7.light	75	10	24.7	29	-14
446.76	632.36	6.2	sp|P01213_1|PDYN_HUMAN.FLPSISTK.+2y6.light	75	10	24.7	34	-14
446.76	535.31	6.2	sp|P01213_1|PDYN_HUMAN.FLPSISTK.+2y5.light	75	10	24.7	30	-14
446.76	316.68	6.2	sp|P01213_1|PDYN_HUMAN.FLPSISTK.+2y6+2.light	75	10	24.7	20	-14
450.77	753.46	6.2	sp|P01213_1|PDYN_HUMAN.FLPSISTK.+2y7.heavy	75	10	24.7	40	-14
450.77	640.38	6.2	sp|P01213_1|PDYN_HUMAN.FLPSISTK.+2y6.heavy	75	10	24.7	34	-14
450.77	543.32	6.2	sp|P01213_1|PDYN_HUMAN.FLPSISTK.+2y5.heavy	75	10	24.7	30	-14
450.77	320.69	6.2	sp|P01213_1|PDYN_HUMAN.FLPSISTK.+2y6+2.heavy	75	10	24.7	20	-14

RT: retention time; DP: declustering potential; EP: entrance potential; CE: collision energy; CXP: cell exit potential, ST1: prefilter

Figure S2. Representative sections of striatum and cerebral cortex used for semi-quantitative neuropathologic analysis. In each section, both astrogliosis and neuronal loss were scored. From the top to the bottom, mild (=1), moderate (=2) and severe (=3) neuropathological changes are shown.

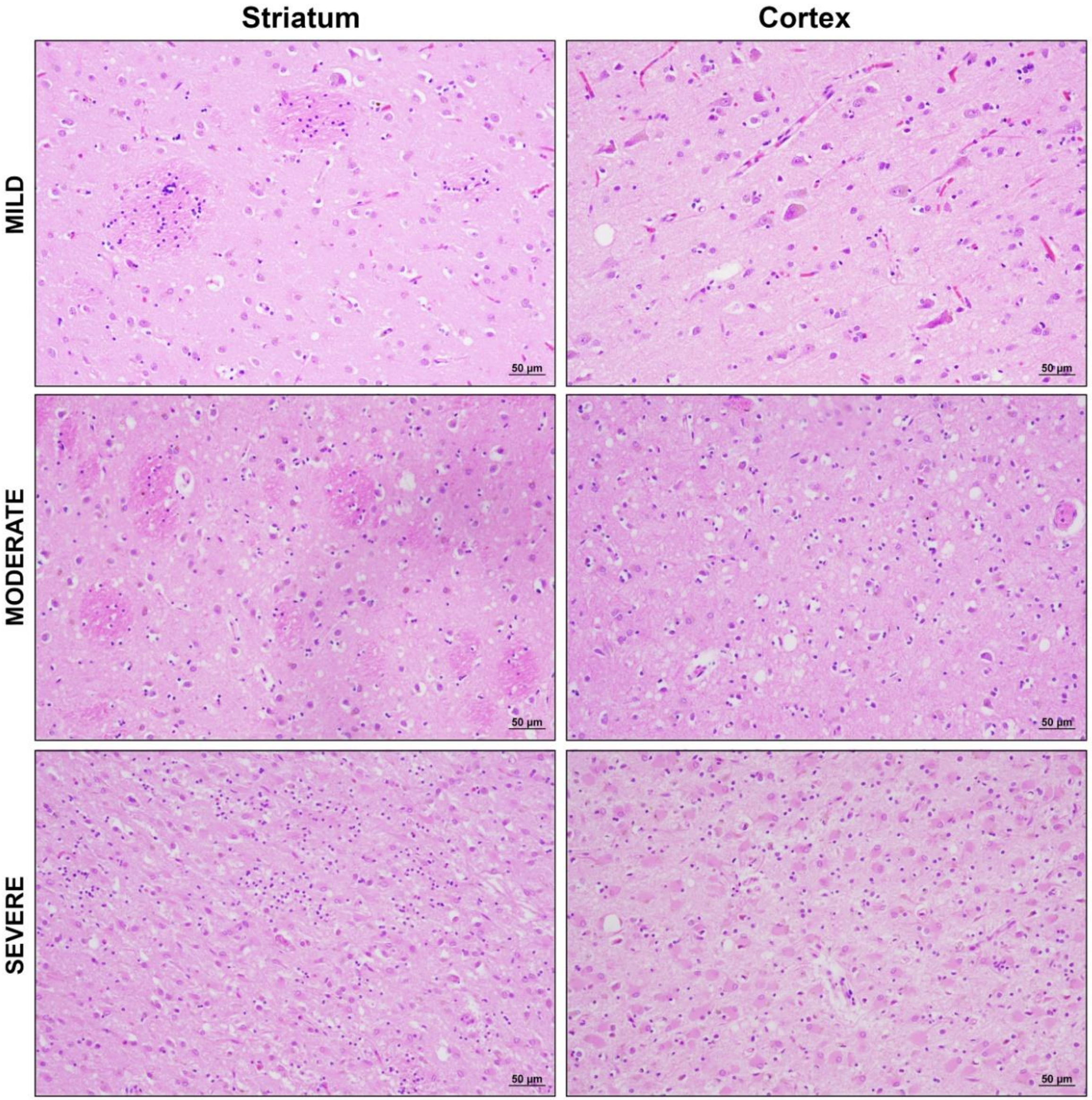

Supplement: Supplementary file 1 [file ijms-23-02051-s001.zip › ijms-1580669-supplementary.pdf]
